# Validity and intra-rater reliability of an Android phone application to measure cervical range-of-motion

**DOI:** 10.1186/1743-0003-11-65

**Published:** 2014-04-17

**Authors:** June Quek, Sandra G Brauer, Julia Treleaven, Yong-Hao Pua, Benjamin Mentiplay, Ross Allan Clark

**Affiliations:** 1 University of Queensland, St Lucia QLD 4072, Australia; 2 Department of Physiotherapy, Singapore General Hospital, Outram Rd, Singapore 169608, Singapore; 3 School of Exercise Science, Faculty of Health Sciences, Australian Catholic University, Fitzroy, Victoria 3065, Australia

**Keywords:** Spine, Whiplash, Inertial monitoring unit, Phone, Cervical range of motion

## Abstract

**Background:**

Concurrent validity and intra-rater reliability using a customized Android phone application to measure cervical-spine range-of-motion (ROM) has not been previously validated against a gold-standard three-dimensional motion analysis (3DMA) system.

**Findings:**

Twenty-one healthy individuals (age:31 ± 9.1 years, male:11) participated, with 16 re-examined for intra-rater reliability 1–7 days later. An Android phone was fixed on a helmet, which was then securely fastened on the participant’s head. Cervical-spine ROM in flexion, extension, lateral flexion and rotation were performed in sitting with concurrent measurements obtained from both a 3DMA system and the phone.

The phone demonstrated moderate to excellent (ICC = 0.53-0.98, Spearman ρ = 0.52-0.98) concurrent validity for ROM measurements in cervical flexion, extension, lateral-flexion and rotation. However, cervical rotation demonstrated both proportional and fixed bias. Excellent intra-rater reliability was demonstrated for cervical flexion, extension and lateral flexion (ICC = 0.82-0.90), but poor for right- and left-rotation (ICC = 0.05-0.33) using the phone. Possible reasons for the outcome are that flexion, extension and lateral-flexion measurements are detected by gravity-dependent accelerometers while rotation measurements are detected by the magnetometer which can be adversely affected by surrounding magnetic fields.

**Conclusion:**

The results of this study demonstrate that the tested Android phone application is valid and reliable to measure ROM of the cervical-spine in flexion, extension and lateral-flexion but not in rotation likely due to magnetic interference. The clinical implication of this study is that therapists should be mindful of the plane of measurement when using the Android phone to measure ROM of the cervical-spine.

## Findings

### Introduction

Cervical range-of-motion (ROM) assessment forms an integral part of physiotherapy evaluation in people with neck-pain by quantifying an important physical impairment [1] and providing potentially useful diagnostic data [2]. In this regard, the cervical range-of-motion device (CROM) [3,4] and single inclinometer are considered the most appropriate clinical measurement instruments. However, the CROM is relatively expensive (US$395) and cumbersome, and the inclinometer although more affordable, has been reported to have inconsistent and inferior validity for cervical lateral-flexion and rotation measurements [5,6].

Advances in smart phone sensor technology have resulted in inexpensive ROM measurement tools with clinical and research potential. Specifically, the smart phone uses an embedded-accelerometer and a magnetometer to detect motion using gravity and the earth’s magnetic field respectively. To our knowledge, only one published study [7] has examined the validity and reliability of the smartphone to measure cervical ROM. Although that study reported some promising findings, it did possess limitations including: a) the criterion reference used (i.e. CROM) did not allow for concurrent testing of the phone, and lacked the sensitivity and precision of a multi-camera three-dimensional motion analysis (3DMA) system, which may have negatively influenced the mostly moderate validity findings; b) no reported effort was made to ensure that movement was well-controlled and along the intended axis of head movement; and c) the examiner was not blinded to the results obtained from the phone and the CROM device, hence error due to reporting bias cannot be ruled out. This may potentially overestimate the validity results. Therefore the purpose of this study was to investigate the concurrent validity and test-retest reliability of an Android smart phone to assess cervical ROM. Our study extends prior research by (i) verifying the validity of the smart phone by concurrently assessing with a 3DMA system, the gold-standard for capturing motion analysis [8], (ii) adding a spirit-level type indicator to the phone application to ensure a pure axis of movement [9] and (iii) blinding the examiner to the results. We hypothesize that the phone will be valid and reliable.

## Methods

### Subjects

Twenty-one healthy individuals (age:31 ± 9.1 years, height: 172.7 ± 8.9 cm, weight:68.5 ± 11.2 kg, male:11) with no reported neck-pain participated. Sixteen participants returned 1–7 days later to assess intra-rater reliability. All participants provided informed consent as outlined by the institution’s Human Research Ethics Committee and all procedures were conducted according to the Declaration of Helsinki.

### Procedures

**Figure 1:**
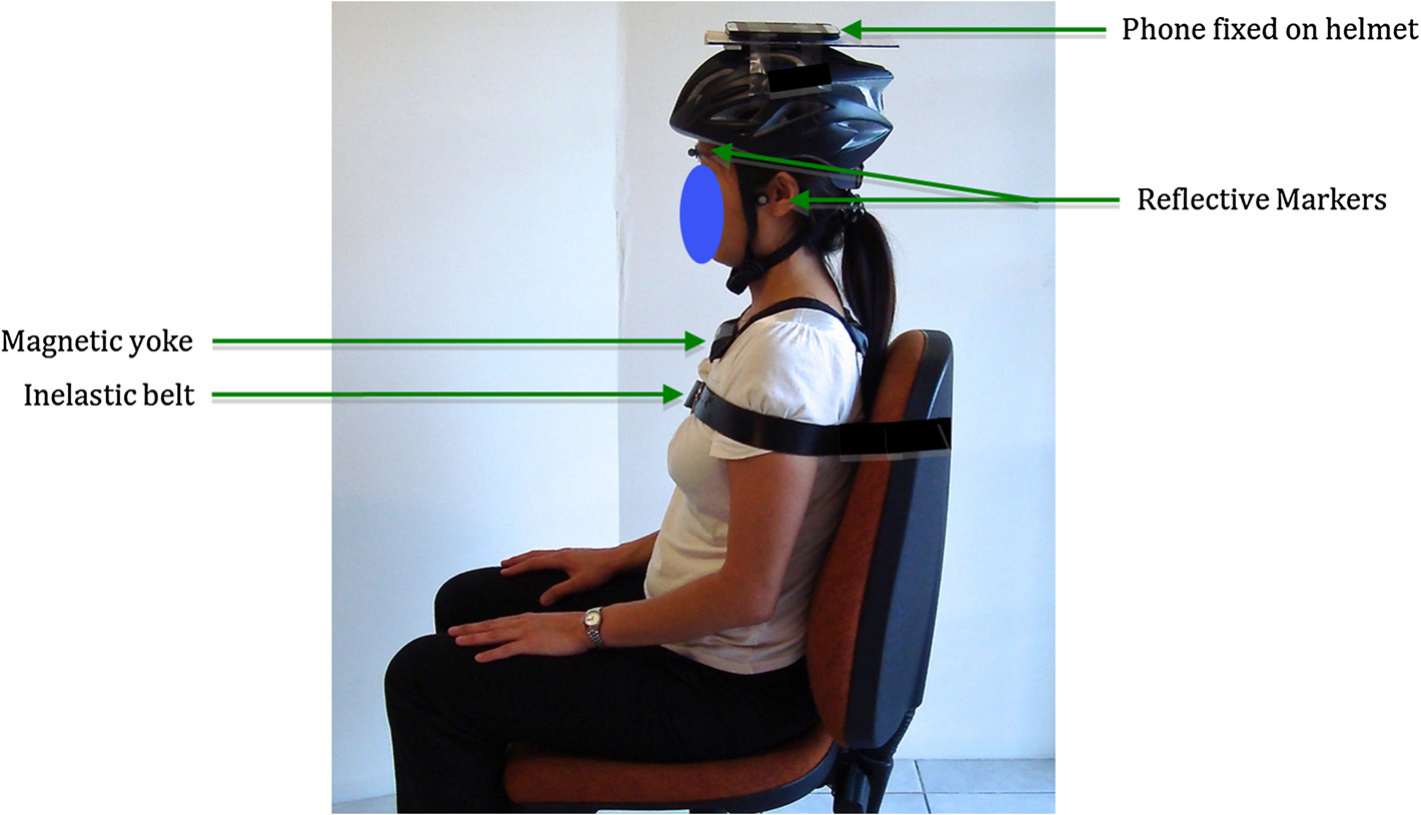
**Experimental setup.** This shows the starting position of the participant and the equipment set-up.

Three reflective markers were located on the following anatomical landmarks: anterior to the tragus bilaterally and on the glabella (Figure 1) for 3DMA analysis. Markers were tracked using VICON Nexus V1.7.1 and a 9-camera VICON MX motion analysis system (VICON, UK). The angle of the head in the three planes was referenced to the laboratory axis, and normalized to the starting neutral position, and was deemed our benchmark reference kinematic data.

All measures were performed with the subject seated in the same high-back padded chair. To ensure minimal contribution from the thoracic spine, the participant was securely strapped across the shoulders to the chair using an inelastic belt (Figure 1: Mulligan Mobilization Belt). An Android 4.0 phone (Samsung Galaxy S3, GT-I9300T) was mounted on a helmet (Figure 1), and the helmet was fastened securely on the patient’s head using an internal adjustable head strap fixed within the helmet. This phone contains a LSM330DLC inertial monitoring unit combining tri-axial accelerometer and gyroscope sensors, and an AKM8975 tri-axial magnetometer.

The following cervical-spine ROM limit measurements were obtained in the same order in all subjects: (i)flexion, (ii)extension, (iii)right-lateral-flexion, (iv)left-lateral-flexion, (v)right-rotation and (vi)left-rotation. The flexion/extension, lateral flexion/extension and rotation axes were measured using the pitch, roll and azimuth angles respectively. Given that cervical rotation values are based on the magnetometer within the phone and the outcome may be influenced by the surrounding magnetic fields, a magnetic yoke was placed around the subject’s neck in an attempt to address this problem. This replicates the use of the CROM, which also uses magnetic fields to determine angles and requires the use of a magnetic yoke.

The patient was instructed to perform each test actively, with manual guidance provided by the examiner to ensure that the movement was along the pure axis of alignment if necessary. Specifically, the examiner determined the end of ROM when a firm resistance was felt. No pain was reported by any subject during the procedure. Three consecutive trials using concurrent measurements from the VICON and the phone were obtained for each movement. The mean value of the three measurements for the first testing day was used to calculate validity, and an inter-day comparison of these mean values was performed to determine intra-rater reliability.

**Figure 2:**
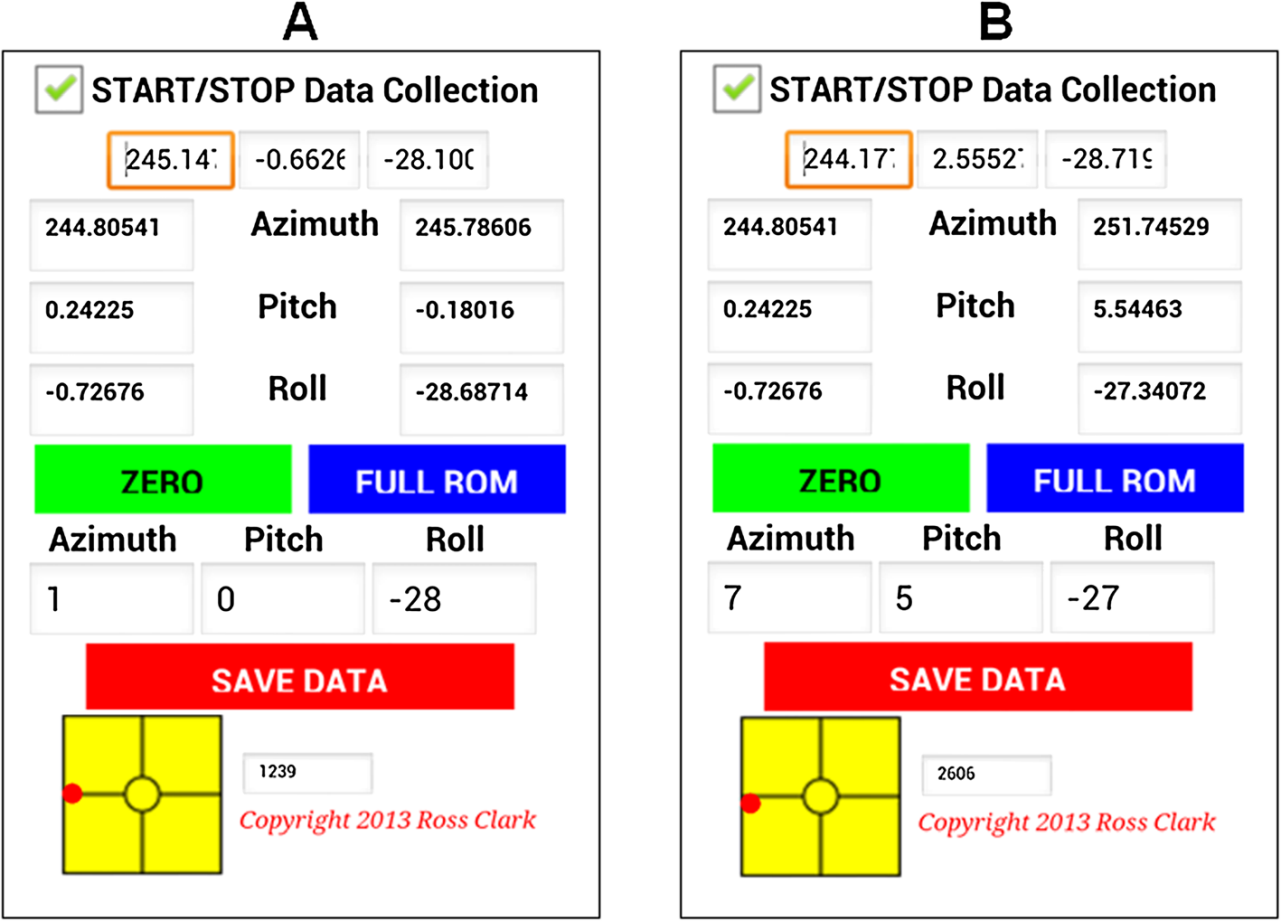
**Comparison of lateral flexion assessment techniques. (A)** Right lateral flexion in a single axis. Note that the red dot is aligned with the horizontal line. **(B)** Right lateral flexion with some cervical flexion. Note that the red dot is not aligned with the horizontal line.

All subjects were assessed by the same examiner (JQ) who has 12 years of clinical musculoskeletal physiotherapy experience. Noteworthy, because it is difficult for the examiner to visually detect when the subject deviates away from the pure movement plane, one of the advantages of this phone application over previous applications [7] was that it included a visual representation of a circular spirit device (Figures 2A and 2B). This enabled the examiner to guide subjects along the desired plane of movement using the real-time visual feedback. This program sampled data at 100Hz using a custom program designed by co-author RC using MIT App Inventor. The standard angle data parsed from the angle calculation performed within the Smartphones operating system was used, indicating that our results are likely to be applicable regardless of the software program used. Two separate examiners were assigned to each device (phone and 3DMA), hence they were blinded to the results of the other device.

### Statistical Analysis

#### *Validity*

Validity was determined from Spearman’s correlation and intra-class correlation coefficient (ICC) in combination with assessment of systematic bias. Bland and Altman plots were constructed to determine the 95% limits of agreement (LoA) between the 3DMA and phone measures [10,11]. Ordinary least products (OLP) regression, which accounts for error in both devices, was used to determine fixed and proportional biases [12]. All calculations were performed as described previously [13].

### Reliability

Intra-rater reliability was determined using intra-class coefficients (ICC [3,3]), and OLP regression to quantify the relationship between sequential measurements for both instruments. ICC was calculated in a 2-way analysis of variance based on absolute agreement. Point estimates of the ICC values >0.75 were considered excellent, 0.4-0.75 modest or <0.4 poor [14]. To estimate measurement error, standard error of measurement (SEM), LOA, and minimal detectable change (MDC) were calculated. Statistical analyses were completed using PASW software V21.

## Results

### Validity

The phone demonstrated excellent concurrent validity for flexion, extension, and lateral flexion ROM based on Spearman’s ρ-values >0.84 and ICC values >0.90, but only modest validity results for left-rotation (ICC =0.53, Spearman’s ρ =0.52) and right-rotation (ICC =0.53) (Table 1). Furthermore, for right- and left-rotation, both proportional and fixed biases were observed (see Table 1 and Additional file 1: Appendix A for the OLP and LOA plots).

**Table 1:** Validity of the phone compared to 3DMA using 3 repetitions of each cervical movement (n = 21)

	**Phone (Mean ± SD)**	**3DMA (Mean ± SD)**	**ICC (3,3)**	Spearman’s ρ*	**95% CI**	**Average systematic bias (CI)**	**Width of 95% LoA**	**% Error**^**†**^	**Prop Bias**^**€**^	**Fixed Bias**^**€**^
Flexion	52.0 ± 8.7	49.9 ± 8.8	0.98	0.99	0.30 to 0.996	None	2.3	2	N	N
Extension	79.3 ± 8.0	80.4 ± 9.9	0.92	0.83	0.80 to 0.97	None	9.6	6	N	N
Right Lateral Flexion^¥^	45.0 ± 7.3	43.0 ± 7.0	0.96	0.93	0.71 to 0.99	None	4.6	5	N	N
Left Lateral Flexion	48.8 ± 8.8	47.8 ± 8.0	0.95	0.92	0.89 to 0.98	None	7.1	7	N	N
Right Rotation	57.1 ± 9.7	70.9 ± 7.2	0.53	0.81	-0.13 to 0.85	-33.7 + 0.31	9.6	8	Y	Y
Left Rotation	65.3 ± 15.1	71.4 ± 5.8	0.53	0.52	-0.60 to 0.80	-71.2 + 0.95	18.6	14	Y	Y

SD = standard deviation; 3DMA = three dimensional motion analysis; ICC = intra-class coefficients; CI = confidence interval; LoA = limits of agreement; Prop Bias = proportional bias; N = no; Y = yes.

*All correlations were p < 0.001 except Left Rotation (p = 0.02).

^¥^Based on n = 20 as markers on one subject were missing.

^†^% Error = 0.5*Width of 95% LOA/[(Mean_Phone_ + Mean_Vicon_)/2].

^€^Proportional and fixed bias were determined from ordinary least products analysis.

### Intra-rater reliability

Intra-rater reliability is presented in Tables 2 and 3. Excellent intra-rater reliability results were observed for both phone and 3DMA measurements in cervical flexion, extension and right- and left-lateral flexion (ICC = 0.82-0.90), but results were poor for the phone in right- and left-rotation (ICC = 0.05-0.33), whilst the 3DMA showed modest intra-rater reliability (ICC = 0.64-0.77). Percentage error values for the phone ranged from 7-40% and 6-9% for 3DMA (Tables 2 and 3). LOA plots are presented in the Additional file 1: Appendix B & C.

**Table 2:** Intra-rater reliability of the phone (n = 16)

	**Phone D1 (Mean ± SD)**	**Phone D2 (Mean ± SD)**	**ICC (3,3)**	**95% CI**	**Sys bias**	**Width of 95% LoA**	**% Error**^**†**^	**Prop bias**^**€**^	**Fixed bias**^**€**^	**SEM**	**MDC**	LOA 2SD (mean diff ±2.1*SDdiff)
Flexion	51.3 ± 7.9	54.9 ± 7.5	0.86	0.38-0.96	N	9.1	9	N	N	3.1	9.2	-12.84 to 5.48
Extension^¥^	79.0 ± 7.6	80.8 ± 7.03	0.82	0.49-0.94	N	11.8	7	N	N	5.0	11.9	-13.67 to 10.3
Right Lateral Flexion	43.5 ± 6.7	44.9 ± 7.0	0.90	0.73-0.97	N	8.3	9	N	N	2.8	8.3	-9.73 to 6.95
Left Lateral Flexion	49.1 ± 8.8	51.2 ± 7.4	0.85	0.57-0.95	N	11.8	12	N	N	4.1	12.2	-14.20 to 10.16
Right Rotation	50.0 ± 17.1	70.5 ± 22.7	0.33	-0.34-0.73	N	48.2	40	N	N	16.4	48.7	-70.79 to 29.81
Left Rotation	64.3 ± 16.3	69.8 ± 15.6	0.05	-1.7-0.67	N	46.8	35	N	N	15.8	46.9	-52.38 to 41.36

SD = standard deviation; ICC = intra-class coefficients; CI = confidence interval; Sys Bias = systematic bias; LoA = limits of agreement; Prop Bias = proportional bias; N = no; Y = yes; SEM = standard error of measurement; MDC = minimal detectable change; diff = difference.

^¥^Based on n = 15 as one subject’s thoracic spine was not well stabilized.

^†^% Error = 0.5*Width of 95% LOA/[(Mean_Phone_ + Mean_Vicon_)/2].

^€^Proportional and fixed bias were determined from ordinary least products analysis.

**Table 3:** Reliability of the 3DMA (n = 16)

	**Phone D1 (Mean ± SD)**	**Phone D2 (Mean ± SD)**	**ICC (3,3)**	**95% CI**	**Sys bias**	**Width of 95% LoA**	**% Error**^**†**^	**Prop bias**^**€**^	**Fixed bias**^**€**^	**SEM**	**MDC**	LOA 2SD (mean diff ±2.1*SDdiff)
Flexion	48.9 ± 7.7	51.9 ± 6.9	0.88	0.54-0.96	N	8.7	9	N	N	3.0	8.91	-14.77 to 8.90
Extension^¥^	79.1 ± 9.9	81.6 ± 9.2	0.88	0.67-0.96	N	10.0	6	N	N	3.4	10.1	-12.69 to 7.56
Right Lateral Flexion	41.7 ± 6.7	42.9 ± 6.9	0.94	0.82-0.98	N	6.8	8	N	N	2.3	6.83	-8.01 to 5.51
Left Lateral Flexion	46.7 ± 7.6	47.1 ± 6.6	0.92	0.78-0.97	N	8.0	9	N	N	2.8	8.32	-8.57 to 7.89
Right Rotation	68.8 ± 5.1	72.7 ± 5.9	0.64	-0.010-0.88	N	10.4	7	N	N	3.6	10.69	-14.45 to 6.81
Left Rotation	70.2 ± 6.7	73.4 ± 6.7	0.77	0.32-0.92	N	11.2	8	N	N	3.8	11.29	-14.35 to 8.03

SD = standard deviation; ICC = intra-class coefficients; CI = confidence interval; Sys Bias = systematic bias; LoA = limits of agreement; Prop Bias = proportional bias; N = no; Y = yes; SEM = standard error of measurement; MDC = minimal detectable change; diff = difference.

^¥^Based on n = 15 as one subject’s thoracic spine could not be effectively stabilized using the experimental technique utilized.

^†^% Error = 0.5*Width of 95% LOA/[(Mean_Phone_ + Mean_Vicon_)/2].

^€^Proportional and fixed bias were determined from ordinary least products analysis.

## Discussion

This study demonstrates that an Android phone can be a valid and reliable tool to measure ROM of cervical flexion, extension and lateral-flexion but not cervical rotation, consistent with previous results [7]. Cervical rotation results cannot be seen as valid and reliable as, although the rotation measurements from the phone showed moderate validity values (ICC = 0.53), the reliability results were poor. Possible reasons for these results are that, in the position tested, both sagittal and frontal measurements rely on the gravity-dependent accelerometers within the phone but the movements in the transverse plane are detected by the magnetometer, which can be adversely affected by any surrounding magnetic fields. This includes equipment such as computers, speakers and some automatic doors, which were all present in the laboratory and may have caused the error observed in this axis. We attempted to overcome this issue using the magnet supplied with the CROM, however our results were still invalid in this axis. This is clinically relevant because strong magnetic fields are likely to be present in many clinical settings and thus rotation ROM assessment using devices that rely on data from the magnetometer cannot be recommended (i.e. rotation in sitting).

Potential reasons for the greater ICC values in the present study compared to previous work [7] are the concurrent measurements and the addition of the spirit level indicator to improve the accuracy of measurement. The latter is especially important because the cervical-spine is a multi-joint structure and susceptible to coupled movements. Furthermore, we minimized measurement errors by standard fixation of the phone on a helmet, compared to the phone being held by hand on the participant’s head in the previous study [7]. This also implies that the phone ought to be mounted on a helmet when it is being used in the clinical setting, and may be considered a limitation of this study. Furthermore, we found that when measuring cervical extension, the combined weight of the helmet and the phone tended to cause the helmet to slip. The examiner overcame this problem by providing adequate support to ensure that the helmet was firmly fixed on the head during the movement.

This study has several other limitations. (i) We did not assess inter-rater reliability and this may potentially limit the applicability of our findings in clinical settings between observers. (ii) We did not include a rigorous warm-up regime to ensure consistent inter-day readiness to perform the movements. While this is unlikely to affect the concurrent validity data (i.e. an increase in range of motion intra-session would be detected by both devices if they are comparable), it may have negatively affected our reliability results. (iii) As a preliminary step to assess the validity and reliability of the Android phone application, all participants were healthy, therefore the results need to be replicated in populations of interest, such as those with neck-pain. (iv) The reliability data of the 3DMA system for the rotation axis was not particularly good, and it is not possible to determine whether this is due to intra-day subject variation (which would provide justification for the poor phone reliability results) or equipment-related measurement error (which would not have affected the phone reliability values).

In summary, this study aimed to establish the validity and intra-rater reliability of an Android phone application to measure cervical-spine ROM and found that cervical flexion, extension and lateral-flexion measurements are both valid and reliable in sitting and may be used in the clinical setting. In contrast, cervical rotation measurements in sitting are neither valid nor reliable likely due to magnetic field interference. We suggest further study to determine whether the phone is valid to measure cervical-rotation in supine, which would use the accelerometer derived angles and is therefore likely to provide more consistent results.

## Competing interests

The authors declare that they have no conflict of interest.

## Authors’ contributions

JQ was involved in the study design, coordination, data collection, statistical analysis and manuscript drafting. SB was involved in the study design, coordination, manuscript drafting and general supervision of the study. JT was involved in the study design, coordination, manuscript drafting and general supervision of the study. PYH was involved in statistical analysis and manuscript drafting. BM was involved in data collection. RC was involved in the study design, application creation, data collection, coordination, manuscript drafting and general supervision of the study. All authors read and approved the final manuscript.

## Supplementary Material

Additional file 1 **Appendix A.** Validity assessment using OLP plots for measurements with proportional bias. Appendix B. Reliability (phone). Normal Bland Altman plots. Appendix C. Reliability (3DMA). Normal Bland Altman Plots. Click here for file
