# Supplementary material for: Validity and intra-rater reliability of an Android phone application to measure cervical range-of-motion
Source: J Neuroeng Rehabil. 2014 Apr 17;11:65. doi: 10.1186/1743-0003-11-65 (PMC4021613; doi:10.1186/1743-0003-11-65)
Supplement: Additional file 1 — Appendix A. Validity assessment using OLP plots for measurements with proportional bias. Appendix B. Reliability (phone). Normal Bland Altman plots. Appendix C. Reliability (3DMA). Normal Bland Altman Plots. [file 1743-0003-11-65-S1.docx]

### APPENDIX A.

### Validity assessment using OLP plots for measurements with proportional bias

###

### Regression-based Bland Altman Plots with proportional bias

### Standard Bland Altman Plots

### APPENDIX B.

### Reliability (Phone)

### Normal Bland Altman Plots

### APPENDIX C

### Reliability (3DMA)

### Normal Bland Altman Plots
